# Death, Dying, and Dignity in the Time of the COVID-19 Pandemic

**DOI:** 10.1089/jpm.2020.0406

**Published:** 2020-09-22

**Authors:** Harvey Max Chochinov, James Bolton, Jitender Sareen

**Affiliations:** ^1^ Department of Psychiatry, Research Institute of Oncology and Hematology, CancerCare Manitoba, University of Manitoba, Winnipeg, Manitoba, Canada.; ^2^ Department of Psychiatry, Psychology, and Community Health Sciences, University of Manitoba, Winnipeg, Manitoba, Canada.; ^3^ Department of Psychiatry, University of Manitoba, Winnipeg, Manitoba, Canada.

If the first casualty of war is truth, the first casualty of coronavirus disease (COVID-19) for patients nearing death is human dignity. Although the pandemic has claimed ∼6800 Canadian lives, ∼60,000 people have died in Canada since the World Health Organization declared the novel coronavirus (COVID-19) outbreak a global pandemic. Given the insidious nature of this virus, care for patients dying of any cause has been distorted in ways previously thought unimaginable. Because of public health restrictions, patients are dying alone. Even for the sickest of sick, whether they are dying in palliative care units, medical or surgical wards, intensive care units, hospices, or long-term care facilities, limited visitation polices are being strictly enforced. The primary contact these dying patients have is with health care providers, with whom touch can only be experienced through layers of latex, eye contact through layers of goggles and plastic shields, and human presence through layers of anxiety, caution, and fear.

A recent systematic review reported that hospitalized patients placed in isolation for medical reasons are more likely to experience depression, anxiety, anger, and loss of self-esteem; health care providers spend less time with them, impacting patient safety with an eightfold difference in adverse events related to supportive care failures.^1^ Other studies have shown that loneliness is a risk factor for mental disorders, such as depression, anxiety, adjustment disorder, chronic distress, and insomnia. Our own research has shown that lack of social support, symptom distress, and not feeling valued or respected can undermine a dying patient's sense of dignity.^2^ Those who are isolated or avoided are especially vulnerable, inclined to feel that they may not only *have* a contagion, but that they *are* a contagion.

Families barred visitation are denied the opportunity to bear witness, advocate for optimal health care, and must forgo final goodbyes. Not having access to their dying loved ones may put families at risk for complicated grief. Not being able to follow a path of least regret leaves many questions unanswered. Did their loved ones receive the best care possible? Were they in pain? What were they thinking about in those final weeks and days of life? Was someone with them when they died? As if that were not enough, the pandemic has meant that families must forgo community rituals of mourning. Funerals have been reduced to graveside services of 5 to 10 people, with live streaming alternatives the only option for those wishing to virtually pay their respects. After an outbreak of severe acute respiratory syndrome-related coronavirus in 2002–2004, the SARS commission final report noted that “those left behind had no opportunity to confront the reality of death and to honor the life of the deceased” (p. 943), with the proviso that “funeral rites must obviously carry lower priority than the need to contain the virulent public health threat” (p. 942). [Funerals and the suffering of families. SARS Commission Final Report; Volume III. Spring of Fear. archives.gov.on.ca/en/e_records/sars/report/v3-pdf/Vol3Chp5v.pdf] These distortions in the process of death and dying foreshadow similar distortions in the process of grieving, and could mean higher risk of various psychiatric morbidities such as depression, anxiety, post-traumatic stress disorder, and suicidal ideation.

The pandemic has seen health care providers being forced to engage in what some have called *impoverished care*, wherein the obligations toward patient care must be weighed against obligations for self-protection and that of one's family. A recent study out of China described the experiences of physicians and nurses with no infectious disease expertise recruited to care for patients with COVID-19.^3^ They reported exhaustion due to heavy workloads and protective gear, fear of becoming infected and infecting others, feeling powerless to handle patients' conditions, accompanied by a sense of being fully responsible for patients' well-being. The pandemic has seen health care providers confronting multiple concurrent deaths, moral distress, helplessness, and burnout. It is too early to say what complications, such as depression, Posttraumatic Distress Disorder (PTSD), substance abuse, or suicide, will manifest.

With dignity under assault, now is the time to be mindful of the ABCD's of dignity-conserving care: A for attitude, B for behavior, C for compassion, and D for dialogue.^2^ Our research has shown that the way dying patients perceive themselves to be seem, reflective of health care provider *attitude* toward them, is the most ardent predictor of maintaining dignity. Clinicians must be mindful that their outlook on patients shapes every clinical encounter. Changing attitudes means changing perceptions. Something as simple as the patient's photograph on their bedside table can remind us of who they are as a person, over and above whatever medical ailment brought them to our attention. The *behavior* component of dignity-conserving care must always be predicated on kindness and respect. Although behavior includes all acts and how one conducts oneself toward the patient, it begins with something as simple as “taking a seat.” A randomized control trial reported that sitting instead of standing at the bedside can have significant impact on patient satisfaction, compliance, and provider–patient rapport, and that patients perceive their provider as being present at their bedside longer when sitting.^4^

Compassion (the C of dignity-conserving care) has been described as *a virtuous response that seeks to address the suffering and needs of a person through relational understanding and action*.^5^ Compassion demands action to mitigate patient suffering. Whether it is bringing a glass of water, helping them change a television channel, finessing their medication, or listening to them (the perfect action for someone yearning to be heard), compassion can obviate our sense of helplessness or therapeutic nihilism. Dialogue (the D of dignity-conserving care) refers to the conversations and communication we have with patients in the service of affirming personhood.

Our research group has developed and tested a simple question coined *The Patient Dignity Question (PDQ)*, which asks patients, “What do I need to know about you as a person to take the best care of you possible?”^6^ In a cohort of 126 palliative care participants, 97% gave permission to have a brief summary of their PDQ response placed on their chart, 99% said they would recommend it to other patients in their circumstances, and 85% felt the information was important for their health care provider to know. Of the 137 health care providers (HPCs) who gave feedback, most indicated that they learned something new from the PDQ, that it affected them emotionally, and heightened their sense of empathy and connectedness with their patients. We also found an association between receptiveness to information gleaned from the PDQ and health care provider job satisfaction, meaning in life, and overall personal empathy. Another tool we have developed, TIME (This Is Me), provides clinicians a somewhat more structured and detailed alternative to the PDQ as a means of eliciting personhood. In a study of personal care home residents, TIME was reported to heighten sense of dignity, change how others might see or appreciate them, and convey what matters to them, including their worries and concerns.^7^

Dying with dignity has been a problem during the COVID-19 pandemic, a problem that transcends national and international borders and one where there is no safe haven or end in sight. Research is needed into dignity-conserving care, ensuring that, wherever patients are dying, dignity does not fall prey to this insidious virus.
